# Mechanisms associated with t(7;12) acute myeloid leukaemia: from genetics to potential treatment targets

**DOI:** 10.1042/BSR20220489

**Published:** 2023-01-30

**Authors:** Denise Ragusa, Liza Dijkhuis, Cristina Pina, Sabrina Tosi

**Affiliations:** 1 College of Health, Medicine and Life Sciences, Division of Biosciences, Brunel University London, Uxbridge, UB8 3PH, U.K.; 2 Centre for Genome Engineering and Maintenance (CenGEM), Brunel University London, Kingston Lane, UB8 3PH, U.K.

**Keywords:** acute myeloid leukaemia, cancer, chromosomal translocation, paediatric

## Abstract

Acute myeloid leukaemia (AML), typically a disease of elderly adults, affects 8 children per million each year, with the highest paediatric incidence in infants aged 0–2 of 18 per million. Recurrent cytogenetic abnormalities contribute to leukaemia pathogenesis and are an important determinant of leukaemia classification. The t(7;12)(q36;p13) translocation is a high-risk AML subtype exclusively associated with infants and represents the second most common abnormality in this age group. Mechanisms of t(7;12) leukaemogenesis remain poorly understood. The translocation relocates the entire *MNX1* gene within the *ETV6* locus, but a fusion transcript is present in only half of the patients and its significance is unclear. Instead, research has focused on ectopic *MNX1* expression, a defining feature of t(7;12) leukaemia, which has nevertheless failed to produce transformation in conventional disease models. Recently, advances in genome editing technologies have made it possible to recreate the t(7;12) rearrangement at the chromosomal level. Together with recent studies of *MNX1* involvement using murine *in vivo*, *in vitro*, and organoid-based leukaemia models, specific investigation on the biology of t(7;12) can provide new insights into this AML subtype. In this review, we provide a comprehensive up-to-date analysis of the biological features of t(7;12), and discuss recent advances in mechanistic understanding of the disease which may deliver much-needed therapeutic opportunities to a leukaemia of notoriously poor prognosis.

## Epidemiology

**Figure 1:**
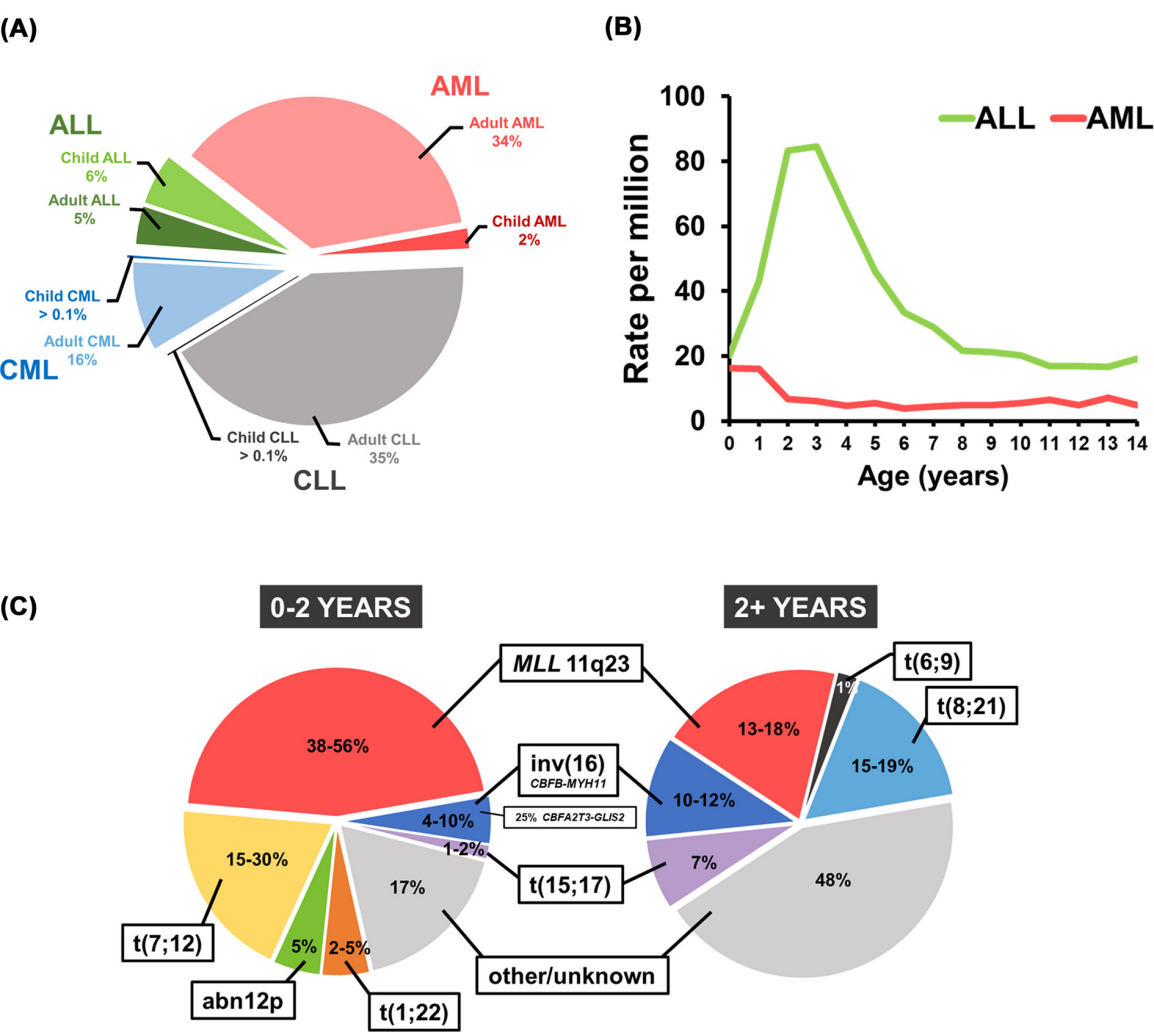
Epidemiology of paediatric leukaemia (**A**) Total cases of leukaemia in the UK by diagnosis (AML, ALL, CML and CLL) and age group (adult or children). (**B**) Incidence of paediatric leukaemia in the U.K. as rate per million by age. The highest incidence of ALL in paediatric patients is between the age of 2 and 4, while AML in paediatric patients manifests mostly between 0 and 2. Data obtained from SEER (Surveillance, Epidemiology, and End Results) Cancer Statistics Review 1975–2017 of the National Cancer Institute (US) and Cancer Research U.K. 2017–2019. (**C)** Frequency of cytogenetic abnormalities in AML cases in infants below the age of 2 (left, 0–2 years) and children above 2 years (right, 2+ years). Specific chromosomal abnormalities [e.g. abn12p, t(7;12), t(1;22), inv(16) *CBFA2T3-GLIS2*] are solely found within a specific age group (<2 years). Modified from Masetti et al. and Bolouri et al. [8,10].

Acute myeloid leukaemia (AML) is a genetically heterogeneous clonal malignancy characterised by uncontrolled proliferation and impaired differentiation of haematopoietic cells of the myeloid lineage. Despite being predominantly a disease of late adulthood, AML also manifests in infants (defined as 0–2 years old) and children (Figure 1A) [1,2]. While overall survival rates have risen over the years, clinical outcomes for paediatric AML patients have remained considerably poorer than in acute lymphoblastic leukaemia (ALL) [2–4]. With approximately 100 cases a year in the U.K., childhood AML is a rare disease, in which relapse, therapy-related toxicity and mortality are major clinical challenges [5]. The age distribution of incidence peaks between 1 and 4 years of age for ALL and between 0 and 2 for AML (Figure 1B), with a rate of 18 per million compared to 8 per million in older children [5,6]. Infant, childhood, and adult AML present age-specific molecular and transcriptional features that have led to their recognition as biologically distinct entities [7–9].

**Figure 2:**
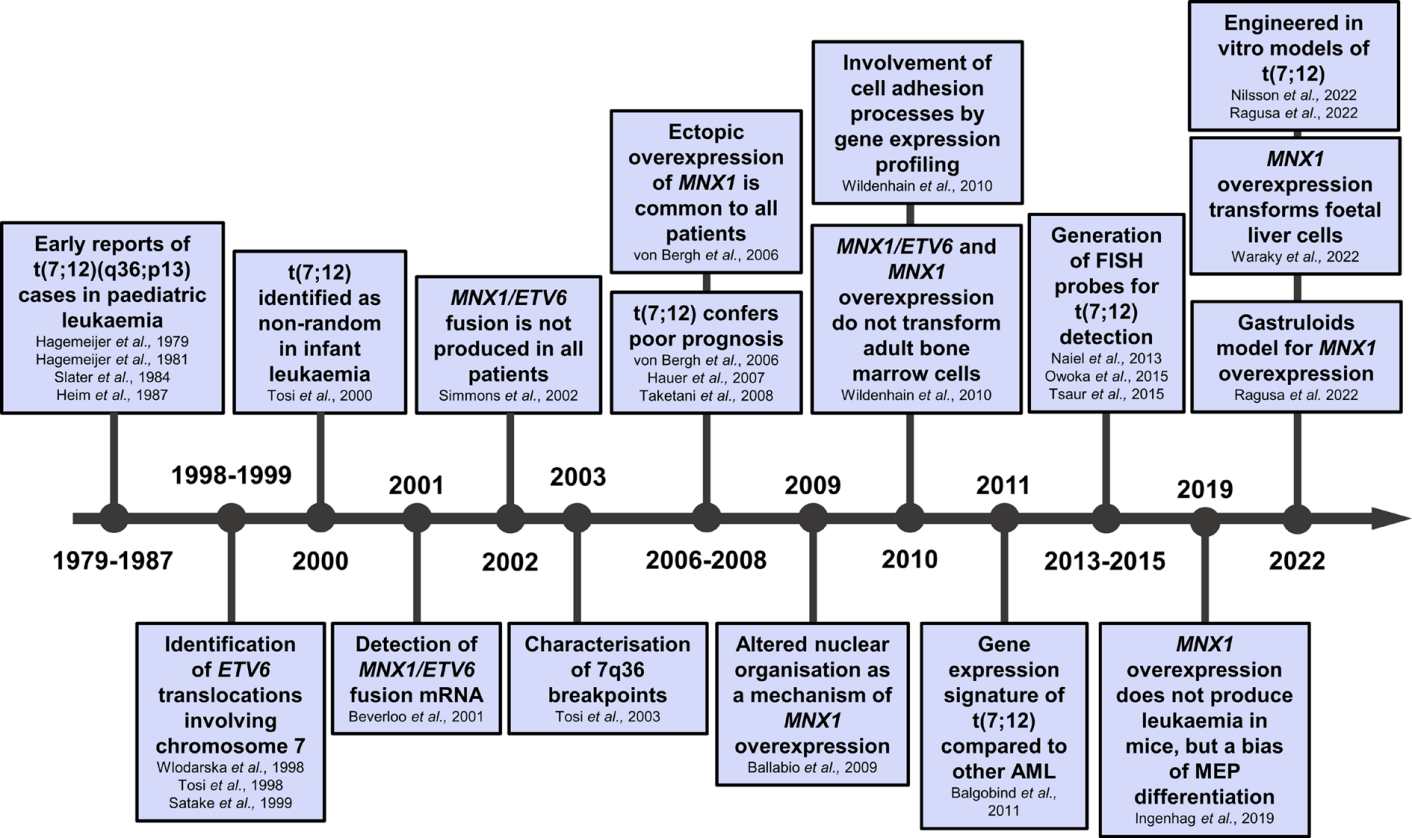
Timeline of key events of research findings on t(7;12) leukaemia Summary of published and preprints on t(7;12)-related literature by year, by inclusion of literature containing keywords ‘t(7;12)’, ‘*MNX1/ETV6*’, or ‘*MNX1*’.

Non-random, recurrent cytogenetic abnormalities, which contribute to the pathogenesis of leukaemia, are an important aspect in the identification of leukaemia subtypes. Cytogenetic features correlate with clinical outcomes and are a major determinant in the risk stratification of patients [11–14]. Certain cytogenetic abnormalities within paediatric AML are restricted to specific age groups (Figure 1C). In infants, the most common abnormalities are 11q23 rearrangements involving the *KMT2A* (*MLL*) gene, and t(7;12)(q36;p13) [10,15], which is exclusive to this age group. Abnormalities of 12p, generally conferring poor prognosis, are also more frequently observed in infants than in older children [16], as well as t(1;22)(p13;q13) which is also exclusive to infants [17]. Normal karyotypes are less prevalent in infants compared to children and adolescents. Similarly infrequent in infants are core-binding factor (CBF) abnormalities, which include inv(16) and t(8;21)(q22;q22) (encoding the *RUNX1-RUNX1T1* fusion), and are usually associated with good prognosis [18]. There are two distinct inv(16) rearrangements, which are differentially prevalent in infants and older children / adolescents. CBF abnormality inv(16)(p13q22) (*CBFB-MYH11*) is characteristic of older children. Infants present with inv(16)(p13q24) (*CBFA2T3-GLIS2*), which is clinically and biologically distinct [19]. In this review, we provide a comprehensive summary of literature on the t(7;12) translocation, which accounts for approximately one third of infant AML cases (Figure 1C), with a historical perspective from the early discoveries to the most recent advances and research strategies (Figure 2).

## Cytogenetic features

**Figure 3:**
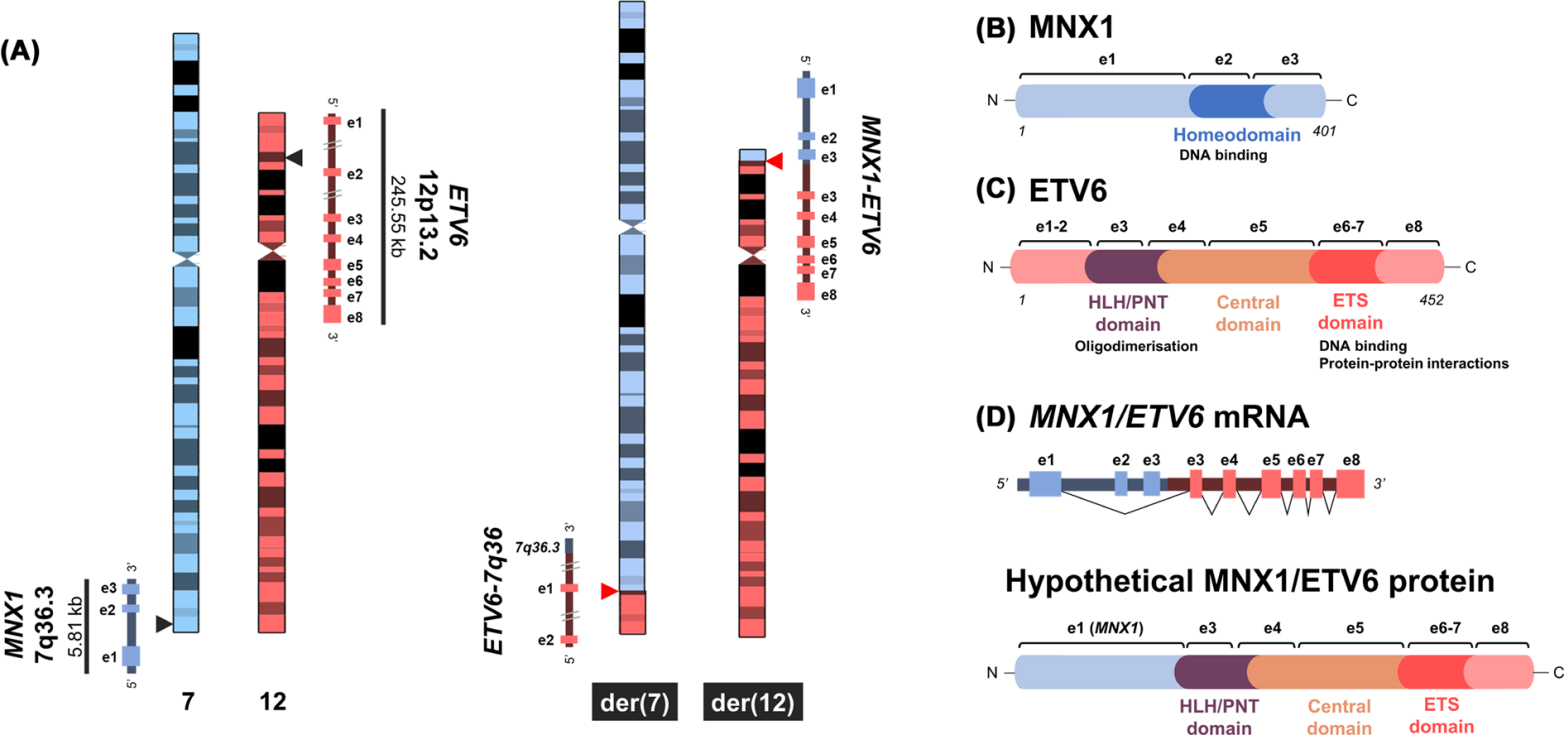
Cytogenetic and molecular features of t(7;12) (**A**) Schematic representation of t(7;12)(q36;p13), showing the non-translocated chromosomes 7 and 12 on the left highlighting the breakpoints at 7q36.3 and 12p13.2 and the structures of the genes involved (*MNX1* and *ETV6*). On the right, the two derivative (der) chromosomes der(7) and der(12), with the resulting fused genomic loci. (**B** and **C**) Protein structures of MNX1 (B) and ETV6 (C), indicating the exons (e) coding for different motifs and their function. (**D**) Schematic representation of the *MNX1/ETV6* fusion which fuses exon (e) 1 of *MNX1* to exon 3 of *ETV6* by a splicing mechanism as shown by black lines. Below, the hypothetical MNX1/ETV6 fusion protein, as described by Beverloo et al. [36], with resulting structural domains.

While early reports of the t(7;12)(q36;p13) translocation can be traced to the 1980s by conventional karyotyping of paediatric AML samples [20–23], it was not until the early 2000s that its cytogenetic features were uncovered in detail [24–27]. As depicted in Figure 3A, t(7;12) is a balanced translocation that produces two derivative (der) chromosomes: der(7) and der(12). The breakpoints map at 7q36.3 proximal to the motor neuron and pancreas homeobox protein 1 (*MNX1*, also known as *HLXB9*) gene on chromosome 7 (Figure 3B), and at 12p13.2 within the ETS Variant Transcription Factor 6 (*ETV6*) gene on chromosome 12 (Figure 3C). While the breakpoint on chromosome 12 is consistently located between exons 1 to 3 of *ETV6*, the breakpoint in 7q36 has been shown to be heterogeneous among patients [28]. Alternative breakpoints have been described in 7q31, 7q32 and 7q35, and some patients also harbour a concomitant deletion of 7q [26,27,29–31]. More complex variants in the form of three-way translocations involving chromosomes 1, 5 and 16 have been reported [32,33]. Table 1 summarises published reports on patients harbouring a classical t(7;12) translocation with breakpoints on 7q36 and 12p13. Table 2 reports examples of karyotypes where (i) chromosomes 7 and 12 are indicated as rearranged and may be involved in deletions, but further analysis is needed to confirm an alternative t(7;12), (ii) the t(7;12) breakpoint on chromosome 7 is indicated as different from q36, (iii) the t(7;12) is part of complex three way rearrangements. Altogether we have included these cases under the umbrella of ‘non-canonical’ t(7;12) rearrangements.

**Table 1:** Reported patients with canonical t(7;12)(q36;p13), defined as balanced translocations with breakpoints on 7q36 and 12p13

Sex	Age (months)	FAB	KARYOTYPE	MNX1/ETV6	Immunophenotype	Reference
M	12	AML	46,XY,t(7;12)(q36;p13)/47,idem,+8	N/A	N/A	[20]
M	14	M4	47,XY,t(7;12)(q36;p13),+8	N/A	N/A	[21]
N/A	7	MDS	46,XX,der(7)t(7;12)(q22;p13)del(7)(q22q36),der(12)t(7;12)(q36;p13)	N/A	N/A	[25]
N/A	20	M5	47,XY,del(7)(q32q35-36),t(7;12)(q36;p13),+19	N/A	N/A	
N/A	N/A	M2	47,XX,t(7;12)(q36;p13),+19/49,idem,+X,+8	N/A	N/A	[47]
N/A	N/A	M2	47,XX,t(7;12)(q36;p13.1),+19	N/A	N/A	
F	5	T ALL	48,XX,t(7;12)(q36;p13),+8,+19	N/A	N/A	[46]
F	5	M1	47,XX,t(7;12)(q36;p13),+19	N/A	N/A	[26]
M	3	M0	47,XY,t(7;12)(q36;p13),+der(19)	Yes	N/A	
M	5	M4	48,XY,t(7;12)(q36;p13),+8,+19	Yes	N/A	
M	5	ALL L2	47,XY,t(7;12)(q36;p13),+19	N/A	N/A	
F	20	AML	47,XX,t(7;12)(q36;p13),+8/48,idem,+19/	No	N/A	
			50,idem,+X,+19,+19/51,idem,+X,+8,+19,+19	N/A	N/A	
F	3	M7	47,XX,t(7;12)(q36;p13),+19	N/A	N/A	[30]
M	4	M1	47,XY,der(7)t(7;12)(q36;p13)del(12)(p13p13),der(12)t(7;12) (q36;p13),+19	N/A	N/A	
F	5	M5	47,XX,t(7;12)(q36;p13),+19/48,idem,+19	N/A	N/A	
F	6	M1	46,XX,t(7;12)(q36;p13)	N/A	N/A	
M	12	RAEB T	46,XY,t(7;12)(q36;p13)	N/A	N/A	
M	18	M3v	47,XY,t(7;12)(q36;p13),+19	N/A	N/A	
M	15	AML	47,XY,t(7;12)(q36;p13),+19	No	N/A	[37]
M	7	AML	48,XY,ins(12;7)(p13;q36;q11.1),+13,+19	No	N/A	
M	19	M0	47,XY,t(7;12)(q36;p13),+19	No	N/A	[28]
M	9	AML	48,XY,t(7;12)(q36;p13),+8,+19	No	N/A	
F	2	M2	47,XX,t(7;12)(q36;p13),+19	Yes	N/A	[29]
M	4	M1	47,XY,del(12)(p13).ish t(7;12)(q3;p13),+19	Yes	N/A	
F	5	M5	47,XX,t(7;12)(q36;p13),+19	No	N/A	
M	18	M3	47,XY,del(12)(p12p13).ish t(7;12)(q3;p13),+19	Yes	N/A	
F	8	M2	47,XX,t(7;12)(q36;p13),+19	Yes	CD34, CD33, CD13, CD38, CD117, CD7, CD56	[41]
F	3	M0	47,XX,t(7;12)(q36;p13),+19	Yes	N/A	[38]
F	48	AML	48,XX,t(7;12)(q36;p13),+8,+19	Yes	N/A	
M	3	ABL	48,XY,t(7;12)(q36;p13),+19,+22	N/A	CD34, CD13, CD33, CD64, CD117, CD7, CD5, CD4	[33]
F	2	M0	47,XX,t(7;12)(q36;p13),+19	N/A	CD34, CD117, CD4, CD7	[42]
F	6	M2	47,XX,t(7;12)(q36;p13),+19	N/A	CD7, CD56	
M	7	AML	48,XY,t(7;12)(q36;p13),+8,+19	Yes	CD34, CD117, CD4, CD7	
F	10	M4	48,XX,+19+22,t(7;12)(q36;p13),inv(16)(p13q22)	N/A	N/A	[32]
N/A	7 (median)	M0	N/A but t(7;12)(q36;p13) confirmed by FISH, +19 in 5/6 patients,	N/A	CD34, CD117 CD7 in 5/6 patients	[44]
		M1		N/A		
		M1		N/A		
		M5a		N/A		
		M5a		N/A		
		ABL		N/A		
M	3	M0	49,XY,14,t(7;12)(q36;p13),+19,+22	No	N/A	[39]
F	3	M2	47,XX,t(7;12)(q3?;p13),+19/48,idem,+13	No	N/A	
F	5	M1	47,XX,t(7;12)(q36;p13),+19	No	N/A	
F	6	M1	47,XX,t(7;12)(q36;p13),del(12)(p11),+19	No	N/A	
F	6	M1	47,XX,t(7;12)(q36;p13),+19	No	N/A	
F	7	M7	47,XX,t(7;12)(q36;p13),+19	No	N/A	
F	8	N/A	50,XX,t(7;12)(q36;p13),+8,+9,+10,+19	No	N/A	
N/A	23	N/A	47,XX,der(7)add(7)(p13)t(7;12)(q36;p13),der(12)t(7;12)(q36;p13), del(17)(q23),+19[20]/46,XX[2]	N/A	N/A	[48]
N/A	2	N/A	47,XX,t(7;12)(q36;p13),+19[15]/47,idem,?inv(17)(p11.2p13)[6]/46, XX[1]	N/A	N/A	
M	10	M7	47,XY,t(7;12)(q36;p13),+19[12]/48,idem,+8[8]	N/A	N/A	
F	77	M1	48,XX,+der(6)t(1;6)(q21;q27),t(7;12)(q36;p13),+19[18] [t(7;12) nuc ish ETV6 sep]	N/A	N/A	
N/A	24	M4	47,XY,t(7;12)(q36;p13),+19[20]	N/A	N/A	

Abbreviation: ABL, acute biphenotypic leukemia.

**Table 2:** Reported patients with non-canonical rearrangements of 7q and 12p, defined as chromosomes 7 and 12 rearrangements other than translocations, t(7;12) breakpoints on chromosome 7 as different from q36, or the t(7;12) as a complex rearrangement

Sex	Age (months)	FAB	KARYOTYPE	Non-canonical feature	MNX1/ETV6	Immunopheno-type	Reference
F	8	M0	47,XX,del(7)(q11.2-21),del(12)(p13), +?	Deletion	Yes	CD34, CD117, CD4, CD7	[24]
M	8	M6	46,XY,der(7)t(7;12)(q32;p13)del(12)(p13)	Deletion; 7q32 breakpoint	N/A	N/A	[27]
			47,idem,+19/47,idem,+8				
F	5	M2	48,XX,t(7;12)(q32;p13),+13,+19	7q32 breakpoint	N/A	N/A	
F	5	T-ALL	50,XX,+6,del(12)(p13),+18,+19,+22	Deletion	N/A	N/A	[26]
F	4	M0	46,XX,t(7;12)(q32;p13)/47,idem,+19	7q32 breakpoint	N/A	N/A	[30]
F	3	M0	46,XX,t(7;12)(q32;p13)/47,idem,+19	7q32 breakpoint	No	N/A	[29]
F	3	M0	47,XX,+19	+19 only	Yes	N/A	
M	18	M3	47,XY,del(12)(p12p13).ish t(7;12)(q3;p13),+19	Deletion; undefined 7q breakpoint	Yes	N/A	
F	23	M7	46,XX,add(7)(q22),del(12)(p12p13)	Deletion	Yes	CD41, CD36,CD13, CD33, CD15, CD7	[31]
M	7	M5a	49,XY,t(5;7;12)(q31;q36;p13),+8,+19,+del(22)(q13)	3-way translocation	N/A	N/A	[33]
M	12	M0	48,XY,t(1;7;12)(q25;q36;p13),+8,+19	3-way translocation	N/A	N/A	
F	4	M2	47,XX,der(16)t(7;12;16)(q36;p13;q12)inv(16)(p11.2q12), +19	3-way translocation	N/A	CD34, CD117, CD4, CD7	[42]
M	4	M2	47,XY,del(12)(q12),+19	Deletion	N/A	CD34, CD117, CD4, CD7	
F	6	M7	47,XX,der(16)t(7;12;16)(q36;p13;q12)inv(16)(p11.2q12), +19	3-way translocation	N/A	N/A	[32]
F	5	MPAL	46,XX, der(7)t(7;12)(q11;p13)del(7)(q11q36)	7q11 breakpoint	N/A	N/A	
N/A	9	M0	47,XX,del(7)(q11.2∼21),del(12)(p13),+?19	7q11 breakpoint	Yes	N/A	[49]

**Figure 4:**
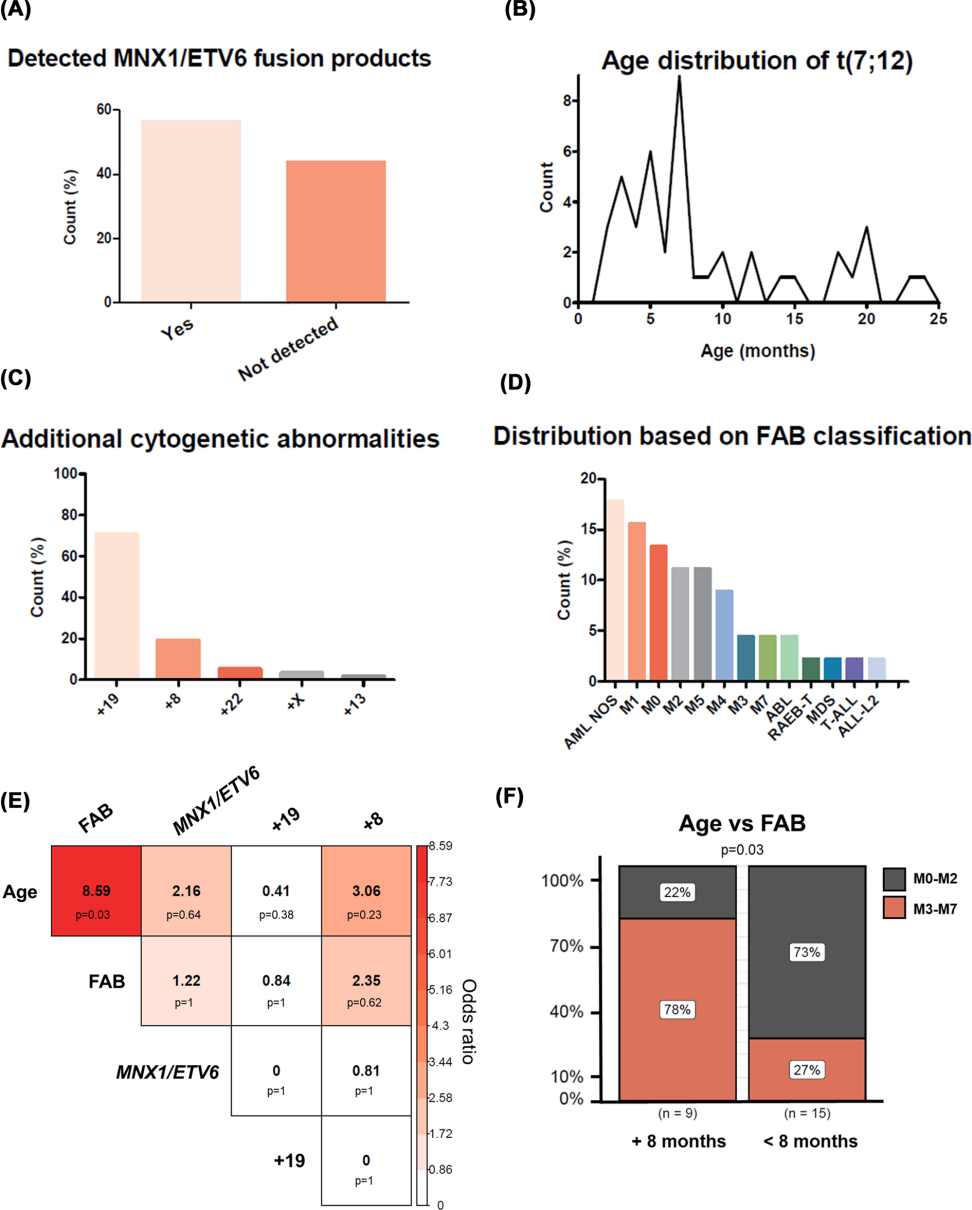
Cytogenetic and clinical characteristics of t(7;12) patients (**A**) Percentage of t(7;12) patient cases where a *MNX1/ETV6* transcript was detected. (**B**) Distribution of age at diagnosis of t(7;12) patients. (**C**) Proportion of additional numerical cytogenetic abnormalities in conjunction with t(7;12). (**D**) Percentage of French American British (FAB) categories of t(7;12) at diagnosis. NOS = not otherwise specified. (**E**) Contingency table of co-occurrence of clinical features of t(7;12) patients showing the odds ratio and statistical significance as *P*-value (threshold < 0.05) obtained by Fisher’s exact test. (**F**) Proportion of t(7;12) patients divided by age (older than 8 months vs younger than 8 months) and the FAB subtype at diagnosis (undifferentiated M0-M2 vs differentiated M3-M7). Statistical significance was calculated by Fisher’s exact test.

Following the cytogenetic characterisation of t(7;12), a major objective of research was to uncover any fusion gene products arising from t(7;12), in line with the pathogenesis of common AML subtypes [34,35]. In 2001, Beverloo et al. [36] published the first report of *MNX1/ETV6* mRNA detected by RT-PCR (Figure 3D). Later reports, however, did not consistently detect fusion transcripts in t(7;12) patients [37–39]. To date, the production of the fusion transcript is estimated to be restricted to half of t(7;12) patients [39,40] (Figure 4A). In t(7;12)(q36;p13), the entire *MNX1* gene is translocated to chromosome 12, upstream of the remainder of the disrupted *ETV6* gene. On the der(7), the 5’ portion of *ETV6* recombines with the genomic portion of 7q36.3. With this arrangement, a functional fusion gene at the mRNA level could only arise from the der(12). Accordingly, a reciprocal fusion has never been shown. The detected *MNX1/ETV6* transcript consists of exon 1 of *MNX1* fused to exon 3 of *ETV6* in-frame. Exons 2 and 3 of *MNX1* are presumably excised via splicing [29,36] (Figure 3D). Out-of-frame isoforms of *MNX1/ETV6* have also been described [41,42], and a translated protein has never been demonstrated. Functionally, the hypothetical resulting fusion would retain the regulatory domains of MNX1 and the HLX/PNT, central and STS domains of ETV6 (Figure 3D). The only common characteristic between t(7;12) patients was later found to be ectopic overexpression of the *MNX1* gene [29,38], which has been used as a surrogate of the disease, as discussed below.

## Clinical features

With a median age at diagnosis of 6 months [39] (Figure 4B), the prevalence of t(7;12) is reported to be between 18-30% of infants with AML. This is possibly an underestimation, as the translocation can go unnoticed using conventional cytogenetic methods. In the late 1990s/early 2000s, advances in molecular cytogenetics, particularly fluorescence *in situ* hybridisation (FISH), allowed a comprehensive visualisation and refinement of the t(7;12) rearrangement [24–26,28]. To date, the most reliable diagnostic approach to detect the translocation is FISH [32,43–45]. Remarkable co-occurring cytogenetic abnormalities are trisomy 8 and/or 19 (Figure 4C), which are deemed to arise as a secondary event conferring proliferative advantage, following the observations of an increased frequency of these aneuploidies at relapse [21,26]. Earlier reports revealed poor prognostic outcomes in infants with t(7;12), with extremely poor survival rates and ineffective treatment by hematopoietic stem cell transplantation [29,31,41]. Event-free survival (EFS) rates were estimated to be 0–14% and overall survival 0–28% by Tosi et al. [40]; however, more recent survival analyses by Espersen et al. [39] described improved prognostic outlooks with 3 year EFS of 43% and 3 year OS of 100% but high rates of relapse.

The clinical phenotype of t(7;12) is predominantly AML, with only rare cases diagnosed as ALL [26,29,46] or as biphenotypic leukaemia [33]. Although t(7;12) is not associated with a specific FAB subtype, the phenotype of blasts often appears poorly differentiated and is therefore often categorised as M0, M1 or M2 (Figure 4D), while some reports also described blasts with a M7 morphology [30,31,39]. Immunophenotypically, blasts express immature markers CD34 and CD117 (c-Kit), CD4 and CD7 T-cell markers, and myeloid markers [33,42]. While extensive analyses of co-occurring clinical features are not available, we explored the correlation between age, FAB subtype, *MNX1/ETV6* fusion, and the two most common numerical abnormalities, trisomies 19 and 8 (Figure 4E). We found a statistically significant association between age and FAB subtype, with younger infants (below the age of 8 months) having a poorly differentiated FAB morphology (M0, M1 or M2) compared to their older counterparts (Figure 4F). Nevertheless, fulfilling the need for detailed clinical studies will help in understanding the biological association between these clinical features.

## Transcriptional landscape

**Figure 5:**
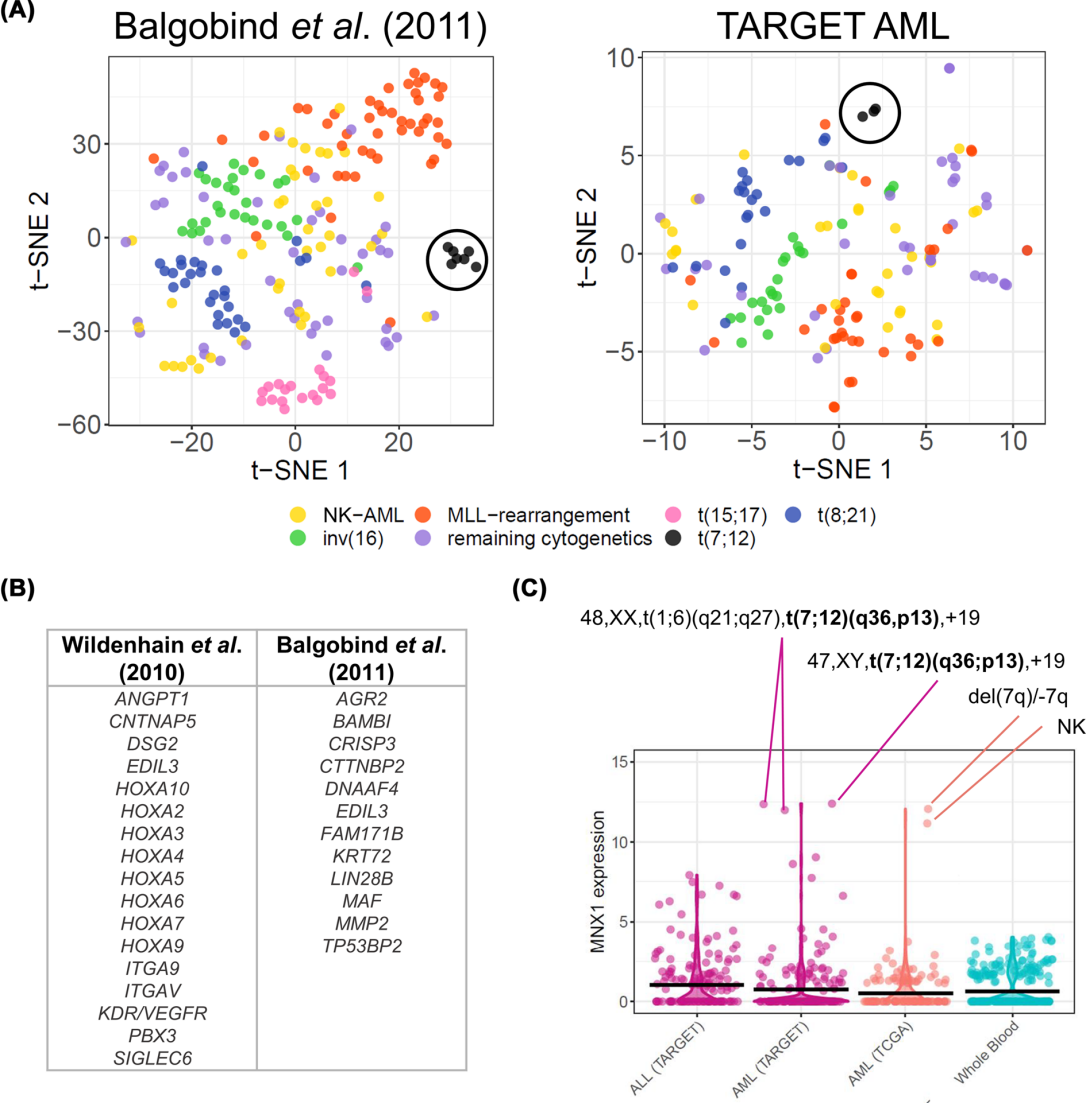
Transcriptional features of t(7;12) patients (**A**) t-SNE plot representing clustering of paediatric AML patients by transcriptional profiles in microarray experiments by Balgobind et al. [50] (left) and in the TARGET RNA sequencing database [48] (right). Patients harbouring t(7;12), circled in black, form distinct clusters from other subtypes, denoting a distinct gene expression signature. (**B**) Distinctive genes in t(7;12) patients derived by microarray analyses by Wildenhain et al. [42] and Balgobind et al. [50]. (**C**) *MNX1* expression levels in the AML cohorts of TARGET (paediatric cancers) and TCGA (adult cancers), and whole blood from GTEx (healthy tissues), determined by RNA sequencing. Expression levels are defined as log2(norm_count+1). The karyotypes for the highest *MNX1* expressors are highlighted. NK = normal karyotype.

Infant, childhood, and adult AML present age-specific molecular and transcriptional features that have led to their recognition as biologically distinct entities [7–9]. The availability of high-throughput genomic tools and public databases enables a comprehensive analysis of the transcriptional landscape of t(7;12). Biological differences between AML subtypes have long been recognised, and microarray gene expression profiling conducted by Wildenhain et al. [42] and Balgobind et al. [50] already revealed a distinct expression pattern for t(7;12) leukaemia. We recapitulate this observation by separate clustering of t(7;12) samples combining publicly-available microarray data and RNA-sequencing (RNA-seq) profiling (Figure 5A).

From these observations, it was apparent that t(7;12)-driven leukaemogenesis differs from other cytogenetic subtypes affecting the same age group. The Wildenhain et al. [42] study clearly highlighted the t(7;12) patient cohort as transcriptionally distinct from patients with *MLL* rearrangements, which are the most common cytogenetic abnormalities in the 0–2 age group [10,15]. Commonly overexpressed genes in the *MLL*-rearranged cohorts, such as the oncogenic *HOXA* gene signature, cofactor *MEIS1*, and *c-MYB* were not up-regulated in t(7;12) patients [42]. In fact, clinical features of *MLL* and t(7;12) infant AML are also distinct, with *MLL*-rearranged AML presenting as M4/M5 morphologies and t(7;12) often exhibiting less differentiated blasts [10,15,33,42]. Wildenhain et al. [42] described upregulation of a number of genes associated with cell adhesion and cell-to-cell interactions in patients with t(7;12) (listed in Figure 5B). Among these, ANGPT1 and KDR/VEGFR2 were highlighted as elements of ligand–receptor signalling pairs, respectively with TIE2 and VEGF, involved in regulation of haematopoietic stem cell (HSC) quiescence and the respective niche microenvironment [51]. The distinctive transcriptional profile of t(7;12) leukaemia is also captured by Balgobind et al. [50], who performed a more comprehensive comparison with 11q23 rearrangements, t(8;21), inv(16) and t(15;17), identifying 13 discriminative genes (Figure 5B). By analysing the Balgobind et al. [50] and TARGET AML datasets, Ragusa et al. [72] derived two gene signatures for t(7;12) patients compared with other paediatric AML subtypes and normal bone marrow. Both gene sets showed enrichment in genes involved in cell adhesion, cellular transport of small molecules, and lipid metabolism, consistent with the findings from Wildenhain et al. [42].

## Biological mechanisms

The biology of t(7;12)-associated leukaemia remains largely unknown, owing to the limited number of patients and the historical lack of research models recapitulating the rearrangement. To date, two main molecular features have been investigated – the *MNX1/ETV6* fusion gene and ectopic overexpression of *MNX1*. While the *MNX1/ETV6* mRNA fusion was only detectable in 50% of cases, ectopic reactivation of the *MNX1* gene is a common molecular feature of t(7;12) patients [29,38]. The unique association of t(7;12) with infants has been proposed to reflect *in utero* origin of the disease [42,52,53], but a candidate cell of origin remains to be identified. The immunophenotype profile of t(7;12) patients exhibiting myeloid and T-cell markers suggested a putative involvement of early T-cell precursors via improper recombination events. However, this hypothesis was disproved by Wildenhain et al. [42], who could not find evidence of V(D)J recombination. A pivotal question in the understanding of (cyto)genetic aberrations in the establishment of the disease is the contribution of the aberration alone or the requirement for additional events. The development of AML follows the cooperative class I and class II mutation model, whereby the full extent of the disease typically requires cooperating events [50]. Nevertheless, as with other types of paediatric/infant AML, additional mutation events are rare. Mutations in *RAD21*, *KIT*, *RAS*, *PTPN11*, and more recently *EZH2*, have been identified in few t(7;12) patients [15,49,54], however comprehensive whole genome sequencing studies are lacking. The co-occurrence of numerical cytogenetic anomalies, most notably trisomy 19 (Figure 4C), may cooperate to the leukaemogenesis of t(7;12) as additional ‘hits’. Nevertheless, the contribution of associated mutations has not been explored.

### *MNX1/ETV6* fusion transcript

Commonly, leukaemia-associated chromosomal translocations (and inversions) result in the generation of fusion oncoproteins or in the activation of proto-oncogenes through the activity of new regulatory elements, which contribute directly to leukaemogenesis [34,35]. The identification of the *MNX1/ETV6* transcript in t(7;12) [36] suggested a conventional fusion-driven leukaemogenic effect, but failure to detect the chimaeric transcript in 50% of cases, and lack of detection of a translated protein brings into question its contributory role to the leukaemogenic process. The transformation potential of an *MNX1/ETV6* fusion transcript was compared with that of the potent fusion oncogene *MLL/ENL*, by viral delivery to adult murine bone marrow HSC, with or without concomitant overexpression of *MNX1* [42]. While the *MLL/ENL* fusion was able to transform HSCs in serial re-plating colony-forming cell (CFC) assays, *MNX1/ETV6* with or without overexpression of *MNX1* did not confer self-renewal capacity. A recent pre-print has also failed to detect leukaemogenic potential of *MNX1/ETV6* fusion-transduced foetal liver (FL) cells in mouse transplantation experiments [55]. However, the authors described an *in vitro* myeloid differentiation bias of cells transduced with the fusion transcript. Significantly, no effects of *MNX1/ETV6* were detected in adult bone marrow (BM) cells [55], hinting at the proposed *in utero* origins of t(7;12) leukaemia. Overall, with a MNX1/ETV6 protein still not identified, the role of the fusion transcript has remained questionable.

### *MNX1* overexpression

The sole unifying molecular characteristic among all t(7;12) patients is the overexpression of *MNX1* [29,38], which has become a defining feature and focal research point of t(7;12) leukaemia. In AML cohorts, ectopic *MNX1* overexpression is almost pathognomonic of t(7;12) and some deletion 7q leukaemias that involve breakpoints proximal to *MNX1* [56] (Figure 5C).

Motor neuron and pancreas homeobox protein 1 (*MNX1*, formerly known as *HLXB9*), is a small homeobox gene of 3 exons coding for the transcription factor HB9, which functions during embryonic development in the formation and differentiation of the pancreas and motor neurons by transcriptional modulation of cell identity programmes [57–61]. Early studies by Deguchi and Kehrl [62] described *MNX1* as highly expressed in human BM CD34+ haematopoietic cells and absent in differentiated blood cells, associating *MNX1* with HSC and progenitors. Later studies, however, produced contrasting results [29,31,63–65]; hence, the physiological expression of *MNX1* in normal blood cells continues to be a topic of debate.

Functionally, Wildenhain et al. [42] demonstrated the inability of *MNX1* overexpression to transform adult BM cells. More recently, Ingenhag et al. [52] investigated the consequences of *MNX1* ectopic overexpression in mouse BM HSCs *in vivo* upon transplantation of irradiated recipients, and observed a block in cell differentiation and the onset of senescence. Engrafted haematopoietic cells lacked mature myeloid cells and B and T lymphocytes, indicating an early block in differentiation. In contrast, the authors detected a significant accumulation of megakaryocyte-erythroid progenitors (MEP), which was further supported by genome-wide expression profiling of engrafted cells which revealed upregulation of genes affiliated with the erythroid lineage. However, overexpression of *MNX1* did not produce malignant transformation in mice. In human CD34+ HSC and progenitors, *MNX1* overexpression resulted in p53/p21-dependent cell cycle arrest and a change in morphology attributable to the induction of senescence, which the authors compared to observations with other known oncogenes, e.g., Myc and RAS in lymphomas or PTEN in prostate tumours. Altogether, the data supported a role for ectopic overexpression of *MNX1* in dysregulation of proliferation and differentiation of normal HSC and progenitors, but could not demonstrate malignant transformation potential in adult mouse and human cells [52].

A recent work in preprint specifically explored the age specificity of *MNX1* activity [55]. Haematopoietic progenitor cells from FL and adult BM were transduced to overexpress *MNX1* and transplanted into immunocompetent and immunocompromised mice. While *MNX1* overexpression had no effect in immunocompetent mice, FL, but not adult BM-transduced cells reportedly initiated a poorly-differentiated myeloid leukaemia in the immunocompromised recipients. Similarly, *in vitro*, the most prominent effects of *MNX1* were observed in FL, specifically skewed myeloid differentiation, and increased CFC capacity and proliferation [55]. This work provides support for a stage-specific role of *MNX1* in leukaemia transformation of foetal cells, and further suggests a facilitating effect of the immature immune system of newborns in propagating the disease.

Transcriptomic analysis of the *MNX1* overexpressing FL captured enrichment of biological functions related to DNA damage, cell cycle, chromatin modelling, and epigenetic modulation [55]. Coherent with Ingenhag et al. [52], Waraky et al. [55] showed induction of DNA damage by *MNX1* overexpression in both FL and adult BM cells. Interestingly, however, the adult but not foetal cells exhibited apoptotic markers, which could explain the age-vulnerability to *MNX1* effects. The authors propose that *MNX1* can affect global chromatin accessibility through interference with MAT2A-mediated availability of methyl groups and imbalance of histone methyl-modifications (H3K4me3 and H3K27me3), but it is unclear how global chromatin regulation links to age-susceptibility to transformation [55]. Ferguson, Gautrey and Strathdee [66] had previously attributed *MNX1* activity in haematopoietic cells to epigenetic dysregulation, and suggested that *MNX1* could act as both tumour-suppressor and oncogene depending on methylation status. The authors found unique hypermethylation of the *MNX1* locus in childhood and adult ALL, and suggested that its demethylated state in AML (as well as in chronic leukaemias) could contribute to transformation [66]. It is possible that the methylation status of the *MNX1* locus and its regulation vary throughout the haematopoietic ontogeny and result in differential susceptibility to methyl group availability and putative self-regulation, with consequences to leukaemogenesis.

Ballabio et al. [38] described an altered nuclear localisation of the loci involved in t(7;12) patient samples. Using FISH, the authors demonstrated that the translocation causes a shift in the nuclear positioning of the translocated *MNX1* locus from the nuclear periphery to the interior. In the nuclei of healthy lymphocytes, *MNX1* is localised at the periphery, and *ETV6* is normally localised in the nuclear interior, therefore the repositioning of *MNX1* into a transcriptionally active (internal) region due to the t(7;12) has been suggested to trigger its expression. Nuclear genome re-organisation due to the chromosomal abnormalities has been shown to affect gene regulation [67,68]; however, it remains unclear whether *MNX1* ectopic expression is a cause or an effect of the nuclear reorganisation. Interestingly, previous nuclear localisation analysis also showed that *MNX1* becomes transcriptionally active during neuronal differentiation of the neuroblastoma SK-N-BE cell line, and this activity is correlated with its relocation towards the nuclear interior [61]. During embryonic development, *MNX1* uses upstream regulatory elements in a tissue-specific manner [69–71], and it is possible that in the context of leukaemia some of these distant regulatory elements are misused. Therefore, the altered genomic context resulting from the t(7;12) rearrangement could play a role in its pathogenesis, presumably through improper contact with tissue-restricted regulatory elements.

## Research models

Detailed mechanistic understanding of t(7;12)-driven leukaemogenesis, and elucidation of the stage-specific consequences of the translocation throughout the ontogeny, require modelling of the translocation itself. To date, t(7;12) leukaemia has been primarily investigated by proxy of *MNX1* overexpression. This has allowed transplantation studies by transgene delivery [52,55]; however, no transgenic mice exist carrying a t(7;12) translocation or haematopoietic overexpression of *MNX1*. Recently, however, we [72] and others [73] successfully recreated the t(7;12) cytogenetic entity in *in vitro* cellular systems by the use of CRISPR/Cas9 genome editing.

Nilsson et al. [73] generated the translocation in human induced pluripotent stem cells (iPSC) using CRISPR/Cas9 delivery via nucleofection, by targeting patient-specific breakpoints on chromosomes 7 and 12. The engineered iPSC line was able to differentiate into all three germ layers with similar efficiency to the parental cells, but upon differentiation into haematopoietic progenitors, t(7;12) iPSC showed a block in erythroid and megakaryocytic differentiation and a myeloid bias. The t(7;12)-carrying iPSC had a proliferative advantage in liquid culture and a higher CFC frequency, but colony output was not sustained through serial replating, with no strong evidence of *in vitro* transformation [73].

We, on the other hand, used the erythro-leukaemia K562 cell line to introduce the translocation by delivery of CRISPR/Cas9 ribonucleoprotein complexes through electroporation [72]. The chosen targets also reflected previously published patient breakpoints [28,37], but were distinct from Nilsson et al. [73]. Phenotypically, the K562-t(7;12) model produced more immature myeloid colonies, which contrasted with the dominant erythroid-like colonies produced by control K562 cells. As the K562 line is already transformed, we were not able to determine transformative potential. Nevertheless, the colony phenotype was retained through serial replating, suggesting self-renewal of a candidate myeloid-biased cell, programmed or selected upon t(7;12) engineering. The t(7;12)-harbouring K562 cells were capable of initiating molecular erythroid differentiation under erythroid culture conditions, but showed a higher magnitude of induction of erythroid marker genes. This observation supported the notion that t(7;12) promotes an earlier erythroid or erythro-myeloid differentiation state, or gives selective advantage to myeloid cells in the absence of differentiation cues [72]. From a mechanistic standpoint, we observed that engineering of t(7;12) could recapitulate the nuclear repositioning of the translocated *MNX1* locus described by Ballabio et al. [38] in t(7;12) patients. By using nuclear localisation analysis on FISH images, we were able to quantify the localisation of translocated and non-translocated chromosomes 7 and 12 within the nucleus, and found that the translocated *MNX1* gene on the der(12) was repositioned towards the nuclear interior.

Both engineered models recapitulate key transcriptional features of t(7;12), importantly the overexpression of *MNX1* and congruent transcriptional profiles of t(7;12) patients [72,73]. An interesting difference between the two models is the generation of the fusion transcript *MNX1/ETV6*, which was not observed in the K562 model [72], but was detectable in the iPSC line at a low expression level [73], potentially capturing differences in generation of a *MNX1/ETV6* fusion in patients that could be sporadic or dependent on the specific underlying translocation breakpoint.

Interestingly, the engineering of t(7;12) posed cell-type-dependent challenges for both groups. Nilsson et al. [73] initially attempted to generate iPSC lines from the BM of a patient harbouring t(7;12), but the translocation was not retained through cloning and reprogramming of leukaemic blasts. Similarly, we [72] introduced the rearrangement in healthy adult CD34+ haematopoietic progenitors and observed a gradual decrease in the frequency of cells harbouring t(7;12) in culture, which were eventually undetectable. The engineered iPSC t(7;12) line by Nilsson et al. [73] was also not transformed by the introduction of the translocation, as determined by the lack of sustained replating capacity. These observations further strengthen the hypothesis that t(7;12) (and *MNX1* overexpression) require a defined cellular background to exert its leukaemic effects [42,52,53,55].

The neonatal presentation of t(7;12)-AML hints at a developmental stage-specific cell of origin. Advances in stem cell biology have harnessed the pluripotency of mouse embryonic stem cells to differentiate into various cell types and organise in embryo-like structures, including gastruloids [74–76]. The latter are 3D cell culture systems initiated from embryonic stem (ES) cells, which self-organise into symmetry breaking polarised structures that mimic *in vivo* gastrulation and can support embryonic tissue and organ formation with temporal and topographical consistency [75,77–79]. In a recent preprint by Ragusa et al. [80], the gastruloid protocol was amended to recapitulate developmental haematopoiesis and served as a model to dissect the cellular basis of t(7;12) infant leukaemia. By overexpressing *MNX1* in this system by lentiviral vector transduction of ES cells, the effects of MNX1 could be pinpointed at a specific temporal window corresponding to the emergence of early CD41+ haematopoietic progenitors. Specifically, *MNX1* overexpressing gastruloids expanded the haemogenic endothelium from which CD41+ haematopoietic progenitors are specified, without affecting CD45+ pre-HSC at later stages of the protocol. Importantly, gastruloids with *MNX1* overexpression gained transformative capacity as observed from serial replating in colony forming assays, further strengthening the developmental specificity of MNX1 leukaemic effects. Analysis of RNA-seq profiles of t(7;12) patients and other AML subtypes against the gastruloids transcriptomics also revealed the specificity of t(7;12) to the early progenitor stage [80]. As a homeobox gene, *MNX1* functions in precise spatio-temporal windows during embryonic development, including axial specification and patterning [59,81]. The gastruloid system is therefore particularly attractive for the study of effects of *MNX1* in a physiologically-relevant context.

## Clinically relevant targets

Despite improvements in therapeutic options and increased understanding of the disease, paediatric AML long-term survival rates are approximately 70% and lag behind the 90% cure rate of ALL patients [2,5,15,18]. In particular, there remains considerable prognostic difference between AML subtypes, with (7;12) infant leukaemia a high-risk group associated with poor clinical outcomes [6,40], despite some improvement in more recent years [39]. The t(7;12) is recognised as an adverse risk cytogenetic abnormality in clinical practice, but it is yet to be included as a distinct entity in the WHO classification of myeloid neoplasms [13,82–84]. Nonetheless, diagnostic tests by FISH are encouraged for screening for t(7;12) in infants [43–45].

Although the unique biological characteristics of infant AML are well acknowledged, therapeutic options of choice are the same as paediatric and adolescent patients [6,85]. Tailored therapy towards biological targets has been successfully employed for well-established leukaemias, classically exemplified by tyrosine kinase inhibitors against BCR-ABL1, or all-trans retinoic acid for PML-RARA [86,87], and more recently extended to FLT3 or IDH inhibitors [88,89]. The repertoire of targeted therapies is expanding for paediatric forms as well, including immunotherapy- or small molecule-based options [90]. As the mechanisms of t(7;12)-mediated leukaemogenesis have remained elusive, the advent of new research tools and models will shed light onto druggable targets specific to t(7;12), for example, by drug screening with the use of cell lines harbouring the rearrangement at the cytogenetic level [72].

Several reports have identified relapse as a major clinical burden of t(7;12) [29,31,41]. Relapse has often been associated with the persistence of leukaemia initiating cells, which are able to evade treatment through modulation of quiescence [91,92]. Specific niche requirements supporting the successful leukaemic effects of t(7;12) have been speculated [42,52,53,65] from the observations of enriched gene expression related to cell adhesion [93–96]. These interactions are crucial during embryonic haematopoiesis to orchestrate mobilisation, migration, and homing of haematopoietic progenitors through the various sites of haematopoiesis [97]. Recent transcriptomics analyses of relapsed AML patients has uncovered new biological pathways associated with the recurrence of disease, and particularly metabolic processes [98]. Interestingly, RNA-seq analyses of the engineered t(7;12) model [72,73] and *MNX1*-overexpressing gastruloids [80] revealed differentially expressed genes related to cellular transport of small molecules, glucose and lipid metabolism, which could be harnessed to identify new druggable targets.

Newer treatment strategies, such as chimeric antigen receptor T cell (CAR-T) therapy are showing considerable success in treating haematological malignancies, particularly lymphoblastic [99]. CAR-T therapy involves the use of autologous T cells that are engineered to selectively attack defined tumour-associated antigens [100]. Newer generations of CAR-T are expanding the targetable CD molecules that are exclusive to malignant cells, which could be adapted to several subtypes, including t(7;12). Target selection for AML has been challenging due to the co-expression of a large number of markers in malignant and healthy haematopoietic cells [101], however high-throughput sequencing of age-specific patients is now allowing the identification of promising candidates [102]. The putative developmental affiliation of t(7;12) leukaemia has the potential to reveal specific antigens which are absent from normal haematopoietic cells at the time of diagnosis, and thus present a credible target of immunotherapy. Extensive immunophenotypic analysis of t(7;12) patients will be needed to confirm the relevance of this approach.

While the role of *MNX1* overexpression is still under investigation, its specificity to the t(7;12) subtype makes it a desirable target. However, the link between *MNX1*'s embryonic activity and haematopoietic malignancies, as well as its expression in healthy haematopoietic cells, remains unclear. Direct transcriptional targets of MNX1 are largely undefined. Using the AML cell line HL-60 transfected to stably overexpress *MNX1*, Wildenhain et al. [65] showed that MNX1 binds and represses PGTER2, the ligand for PGE2, a known regulator of the haematopoietic niche [103]. Down-regulation of PGTER2 is consistently found in transcriptional profiling of *MNX1* overexpression in haematopoietic cells [52,65], suggesting activation of prostaglandin signalling as a candidate therapeutic or disease-modulatory strategy. More targeted therapeutic options could be extrapolated from the preprint by Waraky et al. [55], whereby the epigenetic mechanisms of MNX1 could be diminished by Sinefungin treatment to target the methionine cycle and reset histone methylation. Systematic analysis of direct targets of t(7;12) genome reorganisation and *MNX1* binding, and of their requirement for transformation will reveal new targets for therapeutic intervention.

## Conclusions

AML harbouring t(7;12) is a clinically challenging disease subtype specifically affecting the infant population. Two major molecular features have been investigated to date, namely production of a *MNX1/ETV6* fusion transcript and overexpression of the embryonic gene *MNX1*. Thanks to recent advances in genome engineering technologies, novel murine and human *in vitro* models have been developed. Mechanisms of leukaemogenesis are therefore beginning to be uncovered, including the exact developmental window affected and the role of *MNX1*. Understanding the cytogenetic, molecular, and clinical features of t(7;12) will pave the way towards targeted therapeutic interventions.

## Data Availability

The RNA sequencing datasets analysed in this manuscript are partly based upon data generated by the Therapeutically Applicable Research to Generate Effective Treatments (TARGET) (https://ocg.cancer.gov/programs/target) initiative of the Acute Myeloid Leukemia (AML) cohort phs000465. The data used for this analysis are available at https://portal.gdc.cancer.gov/projects. Microarray data for paediatric AML from Balgobind et al. [54] (under accession number GSE17855) were downloaded from GEO Accession Viewer.

## Abbreviations

ALL: acute lymphoblastic leukaemia
AML: acute myeloid leukaemia
CBF: core-binding factor
CFC: colony-forming cell
EFS: event-free survival
iPSC: induced pluripotent stem cells
